# Prostate apoptosis response protein 4 sensitizes human colon cancer cells to chemotherapeutic 5-FU through mediation of an NFκB and microRNA network

**DOI:** 10.1186/1476-4598-9-98

**Published:** 2010-04-30

**Authors:** Bi-Dar Wang, Christina Leah B Kline, Danielle M Pastor, Thomas L Olson, Bryan Frank, Truong Luu, Arun K Sharma, Gavin Robertson, Matthew T Weirauch, Steven R Patierno, Joshua M Stuart, Rosalyn B Irby, Norman H Lee

**Affiliations:** 1 Department of Pharmacology and Physiology, The George Washington University Medical Center, Washington, DC 20037, USA; 2 Penn State Hershey Cancer Institute, Hershey, PA 17033, USA; 3 Department of Surgery, Penn State College of Medicine, Hershey, PA 17033, USA; 4 Department of Pharmacology, Penn State College of Medicine, Hershey, PA 17033, USA; 5 Biomolecular Engineering Department, UC Santa Cruz, Santa Cruz, CA 95064, USA

## Abstract

**Background:**

Diminished expression or activity of prostate apoptosis response protein 4 (Par-4) has been demonstrated in a number of cancers, although reports on Par-4 expression during colon cancer progression are lacking. An understanding of the molecular events in conjunction with the genetic networks affected by Par-4 is warranted.

**Results:**

Colon cancer specimens derived from patients have significantly diminished expression of Par-4 mRNA relative to paired normal colon. Hence, the functional consequences of reintroducing Par-4 into HT29 colon cancer cells were assessed. Overexpression augmented the interaction of Par-4 with NFκB in the cytosol but not nucleus, and facilitated apoptosis in the presence of 5-fluorouracil (5-FU). Analogous findings were obtained when AKT1 pro-survival signaling was inhibited. Transcriptome profiling identified ~700 genes differentially regulated by Par-4 overexpression in HT29 cells. Nearly all Par-4-regulated genes were shown by promoter analysis to contain *cis*-binding sequences for NFκB, and meta-analysis of patient expression data revealed that one-third of these genes exist as a recurrent co-regulated network in colon cancer specimens. Sets of genes involved in programmed cell death, cell cycle regulation and interestingly the microRNA pathway were found overrepresented in the network. Noteworthy, Par-4 overexpression decreased NFκB occupancy at the promoter of one particular network gene *DROSHA*, encoding a microRNA processing enzyme. The resulting down-regulation of *DROSHA *was associated with expression changes in a cohort of microRNAs. Many of these microRNAs are predicted to target mRNAs encoding proteins with apoptosis-related functions. Western and functional analyses were employed to validate several predictions. For instance, miR-34a up-regulation corresponded with a down-regulation of BCL2 protein. Treating Par-4-overexpressing HT29 cells with a miR-34a antagomir functionally reversed both BCL2 down-regulation and apoptosis by 5-FU. Conversely, bypassing Par-4 overexpression by direct knockdown of *DROSHA *expression in native HT29 cells increased miR-34a expression and 5-FU sensitivity.

**Conclusion:**

Our findings suggest that the initiation of apoptotic sensitivity in colon cancer cells can be mediated by Par-4 binding to NFκB in the cytoplasm with consequential changes in the expression of microRNA pathway components.

## Background

An estimated 400 000 people die of colorectal cancer yearly worldwide [1]. In the US, it is the second leading cause of cancer-related deaths (American Cancer Society, Cancer Facts and Figures 2008). Colon cancer-related mortality often results from metastases, frequently to the liver, that are present at the time of diagnosis. Treatment for metastatic colorectal cancer usually involves a combination of surgery with adjuvant chemotherapy and/or radiation. 5-fluorouracil (5-FU), or a related fluoropyrimidine, has been used as a component of the therapeutic regimen for colon cancer patients for four decades [2-4]. However, despite a combination of 5-FU with other chemotherapeutic agents, the clinical response rate for patients with liver metastases remains 20-39% [5], indicating a need for a more effective regimen.

Targets of chemotherapy include oncogene products such as RAS and SRC, growth factor receptors, and DNA replication machinery. Therapeutic agents consist of nonspecific growth inhibitors such as 5-FU and methotrexate which cause death to any dividing cell, as well as specific targeting drugs. The number of specific targets continues to expand and includes tyrosine kinases for signal transduction, vascular endothelial growth factor for angiogenesis, and growth factors [6-9]. However, the targeting of tumor suppressor genes for therapy poses a different problem. The activity of a tumor suppressor must be induced by replacing or enhancing a missing or inactive protein, respectively, rather than repressing an active protein. The tumor suppressor Par-4 is one such protein being studied as a potential molecular target of cancer therapy [10]. Notwithstanding the potential of Par-4 to be a suitable molecular target, an understanding of Par-4 function in different cancers is warranted. Par-4 is widely expressed in cells, contains a leucine zipper domain through which it interacts with other proteins, and was first isolated from prostate cancer cells undergoing apoptosis [11-13].

The down-regulation of Par-4 has been proposed to be a critical event in tumorigenesis [14]. Par-4 is down-regulated in a number of cancers; namely, endometrial [15], renal cell carcinoma [16], pancreatic [17], and lung cancer [18]. Furthermore, Par-4 has been shown to be inactivated by AKT1 in prostate cancer cells, and a Par-4/AKT1 interaction is widely found in prostate cancer, lung cancer, cervical cancer, as well as in benign prostatic hyperplasia and normal human embryonic lung fibroblasts [19]. The phosphorylation of Par-4 by AKT1 enables the scaffolding protein 14-3-3 to bind Par-4, causing retention in the cytoplasm [19,20].

Overexpressing Par-4 can increase susceptibility of cancer cells to apoptotic agents such as doxorubicin, tumor necrosis factor alpha (TNF-α), and tumor necrosis factor-related apoptosis-inducing ligand (TRAIL) [12,16,21]. While inhibition of Par-4 was shown to reduce sensitivity to exogenous apoptotic stimuli [13,22], Par-4 is essential but not sufficient on its own to sensitize cells to apoptosis [19,23]. Par-4 activity leads to apoptosis via both extrinsic and intrinsic pathways [24-26]. Intrinsic pathways include inhibiting transcriptional regulation by NFκB [25,27,28]. It has been shown that Par-4 inhibits NFκB through multiple mechanisms, such as: (i) Par-4 inhibits RAS- and RAF-induced transcriptional activation of NFκB, without affecting IκB degradation or NFκB nuclear translocation [29]; or (ii) Par-4 binds and sequesters ζPKC [30] (ζPKC phosphorylates IκB kinase which in turn phosphorylates IκB leading to disruption of the NFκB/IκB complex and nuclear translocation of NFκB), enhancing apoptosis initiated by TNFα [27].

Although it has been reported that Par-4 expression can be regulated by nonsteroidal anti-inflammatory drugs in colon cancer cells [31], little has been published on the role of Par-4 in colon cancer, nor has there been an investigation of Par-4 expression as a function of colon cancer progression. We have recently shown that the human colon cancer cell line HT29 becomes sensitized to apoptosis in response to *in vivo *delivery of Par-4 and 5-FU treatment in an animal model [32]. This study was undertaken to examine the mechanism by which Par-4 induces apoptosis in colon cancer cells. We provide evidence for an alternative intrinsic pathway/network involving Par-4 partnering with NFκB in the cytoplasm, disruption of *DROSHA *gene transcription, dysregulation of microRNAs leading to up-regulation of pro-apoptotic and down-regulation of pro-survival targets, and apoptotic sensitization of colon cancer cells to 5-FU.

## Results

### Par-4 expression in colon cancer specimens and cell lines

**Figure 1:**
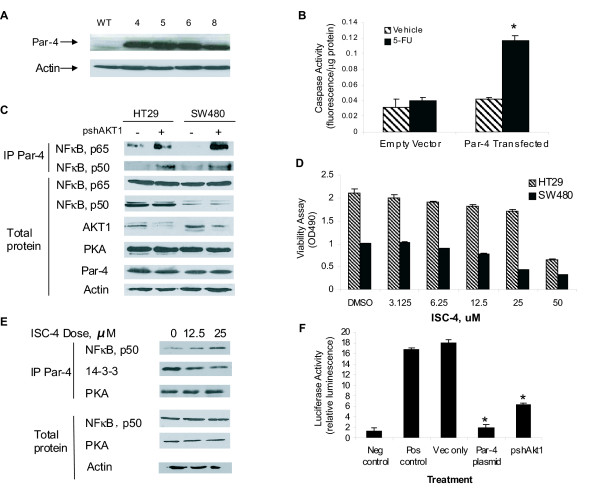
**Par-4 sensitizes colon cancer cells to 5-FU-induced apoptosis and inhibits NFκB activity in an AKT1-dependent manner**. **(A) **Western analysis of Par-4 protein in HT29 cells transfected with empty vector (WT) or pCB6+-*par-4*. Clone 4 was used for subsequent Par-4 overexpression studies. **(B) **5-FU treatment of Par-4- but not empty vector-transfected HT29 cells increases caspase-3 activity. Results are the mean ± S.D. of 4-5 independent experiments. *Significantly different from vehicle (*P *< 0.05, Student's *t*-test). **(C) **AKT inhibition promotes Par-4 binding to NFκB. Native HT29 and SW480 cells were treated with an anti-*AKT1 *shRNA, and lysates without (total) or with Par-4-immunoprecipitation (IP) were immunoblotted for p65, p50, AKT1, PKA, Par-4 and actin. Results are representative of 4-5 independent experiments. **(D) **Inhibition of AKT1 by ISC-4 promotes apoptosis. Native HT29 and SW480 cells were treated for 24 h with ISC-4 and subjected to an MTA viability assay. Decrease in OD reading corresponds to decreased viability. Results are the mean ± S.D. of 4-5 independent experiments. **(E) **Par-4 binding in native HT29 cells treated for 24 h with sub-lethal ISC-4 doses. Lysates without or with Par-4-immunoprecipitation were immunoblotted for p50, 14-3-3, PKA and actin. Results are representative of 4-5 independent experiments. **(F) **Effects of Par-4 overexpression (Par-4 plasmid) and AKT1 inhibition (pshAkt1) on NFκB transcriptional activity in HT29 cells. *Significantly different from empty vector-transfected cells (Vec only) by ANOVA and Tukey post-hoc test (*P *< 0.01). Negative (Neg) and positive controls (Pos) are reporter plasmids without promoter or with SV40 promoter sequences, respectively. Results are the mean ± S.D. of 4 independent experiments.

Quantitative RT-PCR analysis revealed a significant ~3-fold down-regulation of *PAR-4 *mRNA (*P *< 0.05, paired t-test; n = 11 paired samples) in colon cancer patient samples compared with paired normal colon (Additional file 1). To examine the downstream effects of overexpressing Par-4 into colon cancer cells, HT29 cells were transfected with an expression vector containing the *PAR-4 *cDNA. Western blot confirmed Par-4 overexpression in the HT29 transfectants (Figure 1A), resulting in increased sensitivity to 5-FU-mediated apoptosis as defined by caspase activity (Figure 1B). In contrast, empty vector-transfected HT29 cells were resistant to 5-FU treatment (Figure 1B).

### Par-4 binds to NFκB upon inhibition of AKT1

Apoptotic activity of Par-4 is reduced when bound and phosphorylated by AKT1 in prostate cancer cell lines [15,19,33]. Potentially, if AKT1 is inhibited in native (i.e. - no heterologous Par-4 expression) HT29 and SW480 colon cancer cells, then endogenous Par-4 will be activated, sensitizing cells to chemotherapy-induced apoptosis. To examine the consequences of AKT1 inhibition on Par-4 function in human colon cancer cells, AKT1 activity was suppressed by two complimentary approaches: an shRNA against *AKT1 *(pshAkt1) or phenylbutyl isoselenocyanate (ISC-4), a pharmacological inhibitor of AKT [34]. Afterwards, pull-down assays and Western blot analysis were performed on total cell lysates.

Total protein levels of PKA, an activator of Par-4 [28], NFκB p65 and NFκB p50 were unaffected by *AKT1 *knockdown upon transfection of pshAkt1 into native HT29 and SW480 cells (Figure 1C). However, knockdown of *AKT1 *in both cell lines promoted the interaction of Par-4 with the p65 and p50 subunits of NFκB (Figure 1C). Next, native HT29 and SW480 cells were treated with increasing concentrations of ISC-4 and a viability assay was performed. The EC_50 _for ISC-4-mediated apoptosis was 43.34 μM in HT29 cells and 27.67 μM in SW480 cells (Figure 1D). Subsequently, native HT29 cells were treated with vehicle or two non-lethal doses of ISC-4 at 12.5 μM and 25 μM. Par-4 was immunoprecipitated and Western blot analysis on the immunoprecipitates was performed with PKA, NFκB p50, and 14-3-3 antibodies. Results showed that all treatment groups resulted in equal binding of Par4 to PKA (Figure 1E). In contrast, the inhibitor treatment resulted in increased Par-4 binding to NFκB p50 (4-fold maximum) while causing decreased Par-4 binding to 14-3-3 (3-fold maximum), in a dose-dependent manner. (Figure 1E).

### Par-4 down-regulates NFκB transcriptional activity upon AKT1 inhibition

To determine the consequence of increased Par-4 binding to NFκB following *AKT1 *knockdown, an NFκB reporter assay was performed. Given the relative high endogenous expression of both NFκB subunits p65 and p50 in native HT29 cells (Figure 1C), this line was chosen for co-transfection experiments with a plasmid containing the NFκB promoter sequence upstream of the luciferase reporter gene along with empty vector pCB6+ (Vec only), *PAR-4 *cDNA in pCB6+ (Par-4 plasmid) or an shRNA directed against *AKT1 *(pshAkt1). Results show that transfection with the Par-4 plasmid caused a 9-fold reduction in NFκB activity relative to empty vector (Figure 1F). Decreasing *AKT1 *levels with pshAkt1 likewise reduced NFκB activity by 3-fold (Figure 1F).

### Microarray analysis reveals alterations in expression of pro-apoptotic and anti-apoptotic genes in Par-4-transfected cells

**Figure 2:**
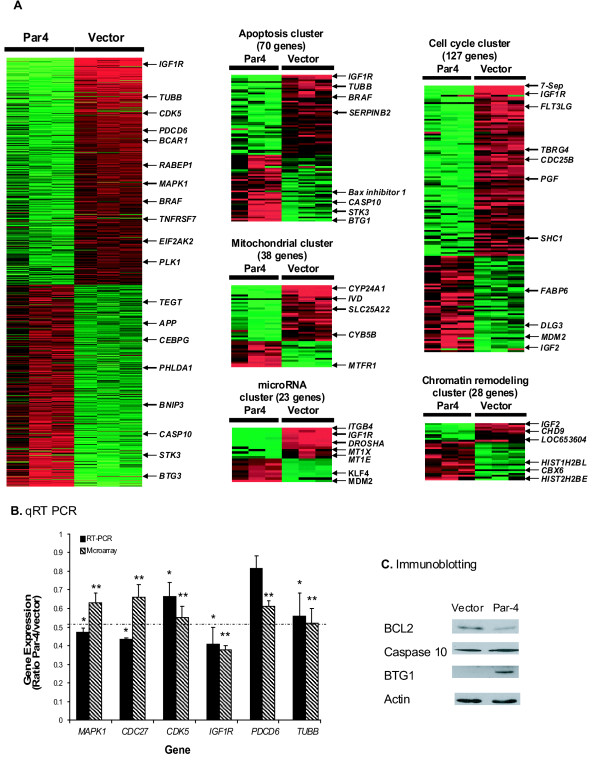
**Global gene expression profiling identifies downstream targets of Par-4-overexpression in HT29 cells**. **(A) **Differential expression of genes in HT29 cells transfected with the *Par-4 *gene versus empty vector. A total of 692 genes were significantly differentially expressed (Par-4-target genes). EASE analysis of Par-4-target genes revealed significant over-representation in GO categories such as apoptosis, cell cycle/proliferation, mitochondrial, chromatin remodeling, and microRNA regulation. Each row in the cluster image represents an individual gene and each column represents an independent hybridization experiment. The relative transcript abundance of each gene is color coded. A red indicates high expression, black indicates intermediate expression and green indicates low expression. **(B) **Validation of selected genes by qRT-PCR. Genes downregulated by Par-4 were validated by qRT-PCR. Results are the mean ± S.D. of 3-4 independent experiments. *,** Significant difference between Par-4-overexpressing cells and empty vector-transfected cells (*P *< 0.05). **(C) **Validation of genes by immunoblot analysis. Caspase 10, BCL2, and BTG1 were assayed by immunoblot analysis. Actin served as loading control. Similar immunoblot results were obtained in 4-5 independent experiments.

To gain further insight into the mechanism of Par-4-mediated susceptibility to apoptosis, we performed genome-wide expression analysis to investigate differences in gene expression between Par-4-overexpressing and empty vector-transfected HT29 cells. A total of 692 Par-4-regulated genes were found to be significantly differentially expressed (ANOVA with 10% or 1% FDR) (Figure 2A; Additional file 2). EASE analysis revealed that the differentially expressed genes were overrepresented in 299 GO categories. Noteworthy were the categories related to mitochondrial function (comprised of 38 genes), apoptosis (70 genes), cell cycle/proliferation (127 genes) and chromatin assembly and disassembly (28 genes). The identity of these downstream target genes along with the direction of their regulation provide a possible mechanism(s) underscoring the apoptotic consequence of Par-4 overexpression in colon cancer cells (see Additional file 3 for representative genes and accompanying biological function/role). Namely, a large number of pro-apoptotic genes were up-regulated and anti-apoptotic genes were down-regulated either directly or indirectly by Par-4 (Figure 2A). Interestingly, the expression of a number of genes associated with and/or regulating the microRNA pathway (e.g. *DROSHA*, *ITGB4*, *IGF1R*, *MT1X*, *MT1E*, *BRAF*) was likewise affected by Par-4 overexpression in HT29 cells (Figure 2A; see Additional file 2 for gene symbols and their corresponding gene names).

### Validation of microarray results by qRT-PCR and immunoblot analysis

Quantitative RT-PCR was used to validate *CDC27*, *PDCD6, TUBB, MAPK1, CDK5 *and *IGF1R *microarray results, genes down-regulated by Par-4 overexpression. Immunoblotting was used to validate Caspase 10 and BTG1 in order to assess whether mRNA regulation was associated with a corresponding change in protein levels. Although levels of *BCL2 *mRNA (a pro-survival gene) were not significantly affected by Par-4 overexpression in HT29 colon cancer cells, we included BCL2 in immunoblotting assays since Par-4 has been shown to regulate *BCL2 *mRNA in prostate cancer cell lines [35]. Actin controls were used for both the immunoblotting and the qRT-PCR analyses. Our results demonstrate near 100% agreement between microarray and qRT-PCR results with the lone exception being *PDCD6 *(Figure 2B), and immunoblotting revealed that up-regulation of *Caspase 10 *and *BTG1 *mRNAs was linked with protein up-regulation (compare Figures 2A and 2C). Finally, immunoblotting indicated that BCL2 protein levels were reduced by approximately 60% despite a lack of change in mRNA levels in HT29 cells overexpressing Par-4, suggesting a genomic mechanism involving translational repression via microRNAs (Figure 2C).

### Overexpressing Par-4 decreases NFκB p65 and p50 occupancy at the promoter regions of NFκB target genes

**Figure 3:**
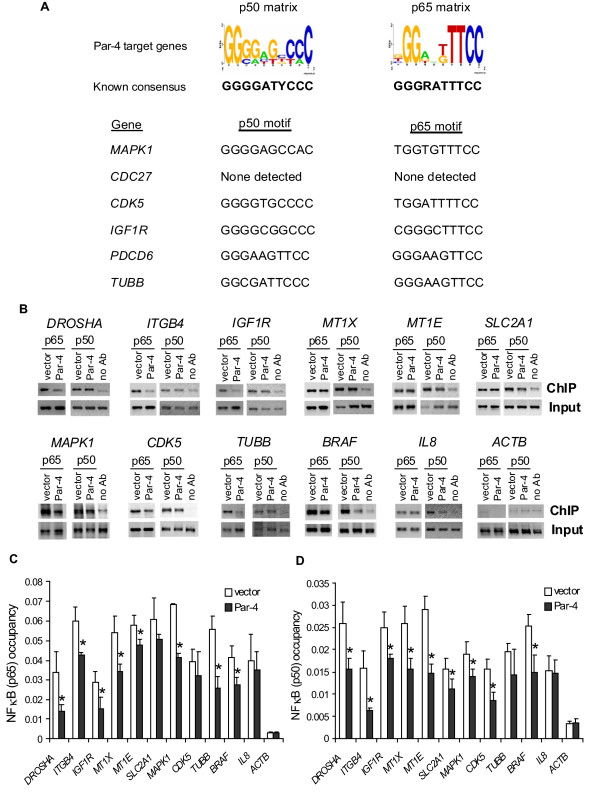
**Overexpressing Par-4 reduces NFκB binding to Par-4 target gene promoters**. (A) Transcription factor binding site analysis of Par-4-target genes for NFκB *cis*-acting sites. Response element sequence logos for NFκB family members p50 and p65 were generated from matrices in the TRANSFAC database using WebLogo (top panel) [76]. The height of each nucleotide base indicates overall conservation at that position. Representative sequence motifs identified by *tffind *for *MAPK1*, *CDC27*, *CDK5*, *IGF1R*, *PDCD6 *and *TUBB *genes are provided in the bottom panel. **(B) **ChIP-qPCR in HT29 cells transfected with empty vector pCB6+ or Par-4 expression vector. Chromatin DNA from p50- or p65-immunoprecipitates (ChIP), no antibody control (no Ab), or starting chromatin (Input) was amplified using quantitative PCR with primers for promoter regions of *DROSHA, ITGB4, IGF1R, MT1X, MT1E*, *SLC2A1, MAPK1, CDK5, TUBB, or BRAF*, and with primers for *IL8 *(positive control) and *ACTB *(nonspecific control). **(C) **and **(D) **PCR products were quantified by measuring 2^(Ct Input - Ct ChIP)^, and the ratios of ChIP-to-input signals were used to yield relative NFκB p65 and p50 enrichment values. Averages and standard deviations from 6 independent ChIP experiments are plotted. *Significantly different NFκB occupancies at target genes between Par-4-overexpressing cells and empty vector-transfected cells (*P *< 0.05). See Additional file 2 for gene symbols and their corresponding gene names.

**Figure 4:**
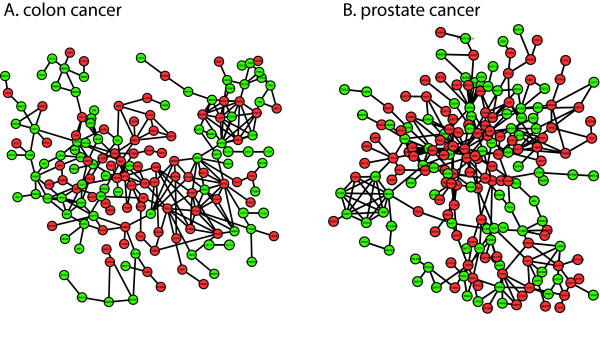
**Recurrent co-regulated gene networks in patient cancer samples**. The set of Par-4-modulated genes with NFκB sites identified in HT29 colon cancer cells were analyzed in patient cancer sample microarray data to define recurrent co-regulated gene networks. (**A**) Colon cancer recurrent co-regulated network of 240 genes with 311 links. (**B**) Prostate cancer recurrent co-regulated network of 276 genes with 367 links. A subset of Par-4-modulated genes identified in HT29 cells can also be found in a recurrent co-regulated network in prostate cancer samples. This suggests that Par-4 co-regulates overlapping genes with NFκB sites in both colon and prostate cancer samples. Red and green nodes indicate genes that were found to be up- or down-regulated by Par-4 in HT29 cells, respectively.

The vast majority of genes (687 out of 692) differentially expressed in Par-4- versus empty vector-transfected HT29 cells was identified by promoter analysis as putative NFκB target genes (see Figure 3A and Additional file 4). The clinical relevance of the Par-4-modulated genes with NFκB binding sites can be illustrated by our recurrent co-regulated network analysis of public microarray data derived from colon cancer patient specimens where 240 (including the microRNA pathway/regulation, pro-apoptotic and pro-survival genes) out of 687 genes were interconnected by 311 links (Figure 4A). Remarkably, the genes comprising the recurrent colon cancer network were also constituents of a recurrent network for prostate cancer (Figure 4B) but not breast cancer (data not shown). Co-regulated gene networks from a single study (i.e. non-recurrent) have been used to gain transcriptional organizational insight into a number of cancers, such as lung, breast and liver tumors [36,37]. The advantage to our recurrent analysis is the inclusion of multiple independent microarray studies from different laboratories to define robust gene links associated with cancers.

Next, we performed ChIP-qPCR assays in Par-4- versus empty vector-transfected HT29 cells to validate the occupancies of both p65 and p50 NFκB subunits at the predicted target binding sites. All target genes selected for ChIP-qPCR assays were identified by microarray analysis as down-regulated in response to Par-4 overexpression and represented a spectrum of gene ontologies, including apoptosis, microRNA pathway/regulation and cell cycle (Figure 2A, Additional file 2). Moreover, the selected genes were shown to be constituents of the colon cancer recurrent network (Figure 4A). The gene *IL8 *was previously identified to be transcriptionally activated by NFκB targeting [38] and was used as a positive control. The housekeeping gene *ACTB*, was used as a negative (nonspecific) control in the NFκB ChIP-qPCR experiments. As expected, ChIP assays in empty-vector-transfected HT29 cells demonstrated that both p65 and p50 were bound to the *IL8 *promoter (Figure 3B, C and 3D). Furthermore, the ChIP-qPCR assays revealed that both NFκB p65 and p50 were significantly enriched at predicted binding elements on promoter regions of *MAPK1*, *CDK5*, *SLC2A1*, *IGF1R, TUBB, BRAF*, *DROSHA, ITGB4, MT1X *and *MT1E*, when compared with the no antibody ChIP-qPCR control and the nonspecific occupancy at *ACTB *(Figure 3B, C and 3D). In comparison to the empty vector-transfected cells, the binding of NFκB p65 and/or p50 at target sites was significantly decreased (*P*-value < 0.05) in the Par-4-transfected cells (Figure 3C, and 3D). *IL8 *gene expression was not associated with Par-4 regulation, and consequently there was no corresponding change in NFκB binding between empty vector- and Par-4-transfected cells. These results indicate that Par-4 modulates NFκB binding to downstream target genes in colon cancer cells.

### Par-4 partners with NFκB in the cytoplasm

**Figure 5:**
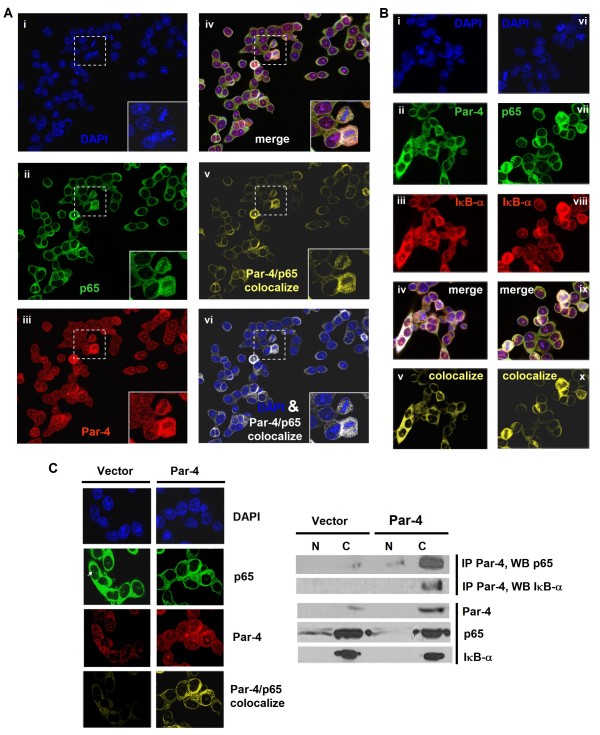
**Interaction of Par-4 with NFκB and IκB in the cytoplasm of Par-4 overexpressing cells**. (**A**) Confocal immunofluorescence of Par-4-transfected HT29 cells triple-stained for Par-4, NFκB p65 and DAPI. DAPI (blue, panel i), p65 (green, ii); Par-4, (red, iii); merge (iv) of Par-4 (red), p65 (green), Par-4/p65 colocalization (white) and DAPI; Par-4/p65 colocalization (yellow, v); overlay of Par-4/p65 colocalization (white) and DAPI (vi). Magnified images (dashed rectangles) inserted in bottom right of panels. (**B**) Confocal immunofluorescence on Par-4-transfected HT29 triple-stained for Par-4, IκB and DAPI (panels i-v) or triple stained for p65, IκB and DAPI (vi-x). DAPI (blue, i and vi); Par-4 (green, ii); p65 (green, iv); IκB, (red, iii and viii); merge (iv) of Par-4, IκB, Par-4/IκB colocalization (white) and DAPI; merge (ix) of p65, IκB, p65/IκB colocalization (white) and DAPI; Par-4/IκB colocalization (v); p65/IκB colocalization (x). (**C**) Par-4 overexpression increases Par-4 associations with NFκB and IκB, and inhibits NFκB translocation. Left panel: Confocal immunofluorescence in empty vector- and Par-4-transfected HT29 cells stained for DAPI (blue), NFκB p65 (green), Par-4 (red) and Par-4/p65 colocalization (yellow). The arrow indicates higher nuclear p65 signal in empty vector-transfected HT29 cells. Right panel: Co-immunoprecipitation of Par-4 followed by Western blot analysis of NFκB p65 and IκB in nuclear (N) and cytoplasmic (C) fractions of empty vector- and Par-4-transfected HT29 cells. The upper panels depict co-immunoprecipitation with Par-4 antibody followed by immunoblotting with p65 and IκB antibodies. Bottom panels depict protein levels of Par-4, p65 and IκB before co-immunoprecipitation.

Par-4 was shown to interact with NFκB in total cell lysates following *AKT1 *knockdown (Figure 1C). In addition, overexpression of Par-4 significantly reduced NFκB occupancy on target gene promoters (Figure 3B, C, and 3D) and decreased NFκB transcriptional activity in a reporter assay (Figure 1F). It is notable that Par-4 overexpression does not enrich for Par-4 at NFκB-bound genes as defined by Par-4 ChIP-qPCR (data not shown). Consequently, we wanted to test the possibility that a direct interaction of Par-4 with NFκB in the cytoplasm, thus inhibiting nuclear translocation of NFκB, may be responsible for the observed gene expression changes. Alternatively, Par-4 may behave as a transcriptional modulator by interacting with NFκB in the nucleoplasm. Colocalization of Par-4 with NFκB p65 and Par-4 with IκB was tested using confocal scanning laser immunofluorescent microscopy and colocalization analysis in Par-4-overexpressing HT29 cells. Figure 5A depicts the immunofluorescent signal for Par-4 (red) which was distributed in both the cytoplasm and nuclei, while the signal for NFκB p65 (green) was mainly distributed in cytoplasm. The colocalized signal (yellow) for Par-4 and NFκB p65 was found almost exclusively in the cytoplasm, with little to no colocalization signal observed in the nucleus (Figure 5A). Similar restricted cytoplasmic colocalization was seen for Par-4 and IκB (Figure 5B). The cytoplasmic colocalization of NFκB p65 and IκB served as an immunostaining control (Figure 5B).

To provide further confirmation of protein-protein interactions among Par-4, NFκB and IκB, we performed subcellular fractionation, co-immunoprecipitation and Western blot analyses in empty vector- and Par-4-transfected HT29 cells. Prior to co-immunoprecipitation experiments with Par-4 antibody, empty vector-transfected cells displayed a prominent p65 signal in the nucleus, whereas Par-4 overexpression clearly reduced this signal in the nucleus (Figure 5C, p65), suggesting that Par-4 overexpression blocks nuclear translocation of NFκB in colon cancer cells. Moreover, co-immunoprecipitation in subcellular fractions followed by Western blot analysis demonstrated that Par-4 associations with p65 and IκB were significantly enriched in the cytoplasm upon Par-4 overexpression (Figure 5C, IP Par-4 and WB with p65 or IκB). Consistently, the cytoplasmic Par-4/p65 colocalized signal was enhanced by Par-4 overexpression (Figure 5C, Par-4/p65 colocalize panel). Collectively, the confocal colocalization microscopy and co-immunoprecipitation findings are consistent with a model of Par-4 interacting with NFκB p65 and IκB in the cytosol, and the resulting partnerships presumably restricting NFκB translocation and subsequent target gene regulation in the nucleus.

**Figure 6:**
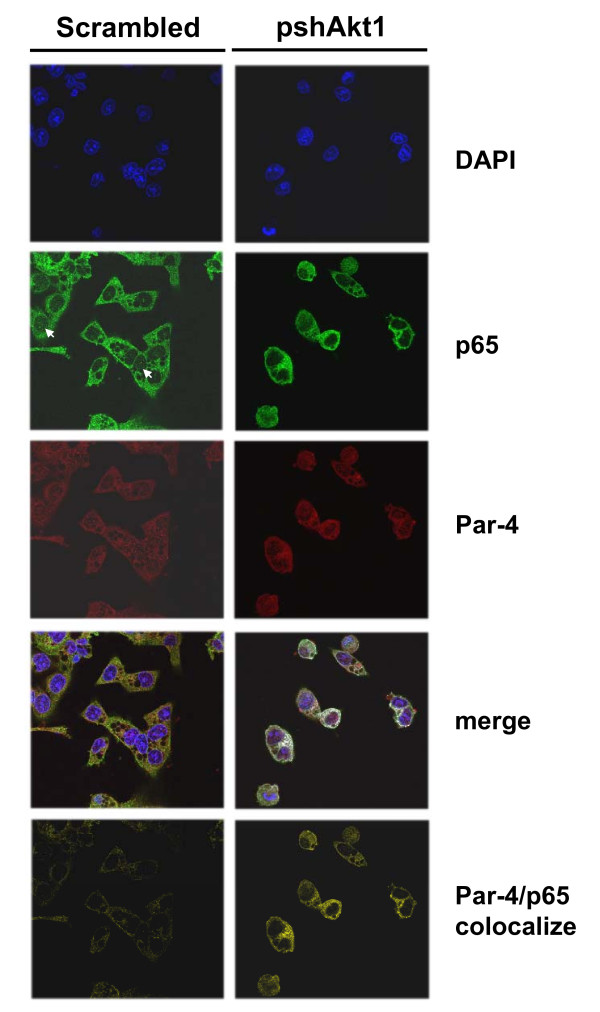
**Increased cytoplasmic Par-4/p65 interaction upon AKT1 inhibition in colon cancer cells**. Native HT29 cells were transfected with scrambled control shRNA (scrambled) or anti-*AKT1 *shRNA (pshAkt1) for 48 h. Cells were triple-stained for Par-4, NFκB p65 and DAPI for confocal immunofluorescence microscopy and colocalization analysis. DAPI (blue); NFκB p65 (Alexa 488, green); Par-4 (Alexa 594, red); merge of Par-4 and p65 for colocalization (white) and DAPI staining (blue); Par-4/p65 colocalization (yellow). Arrows indicate the nuclear p65 signals in native HT29 cells.

Next, we tested whether *AKT1 *knockdown would affect the amount or degree of Par-4 and p65 colocalization. Confocal microscopy was performed in native HT29 cells treated with scrambled control (scrambled) or shRNA against *AKT1 *(pshAkt1). In scrambled-treated HT29 cells, there was a weak basal signal indicative of a low amount of p65/Par-4 interaction in the cytoplasm (Figure 6; Par4/p65 colocalize panel for scrambled). This finding is consistent with results depicted in Figures 1C (first row of blots for native HT29) and **5C **(Par-4/p65 colocalize panel for empty vector-transfected HT29). Following *AKT1 *knockdown, the cytoplasmic Par-4/p65 colocalized signal was significantly enhanced (Figure 6; Par4/p65 colocalize panel for pshAkt1). Moreover, the nuclear p65 signal tended to be reduced upon *AKT1 *knockdown in native cells (Figure 6; p65 panels for scrambled and pshAkt1), analogous to the confocal and Western results found in Par-4-overexpressing versus empty vector-transfected cells (Figure 5C). Taken together, these results suggest that inhibition of *AKT1 *extensively augments Par-4/p65 interactions in cytosol and consequently blocks nuclear translocation of p65.

### Par-4 overexpression alters DROSHA and microRNA expression

**Figure 7:**
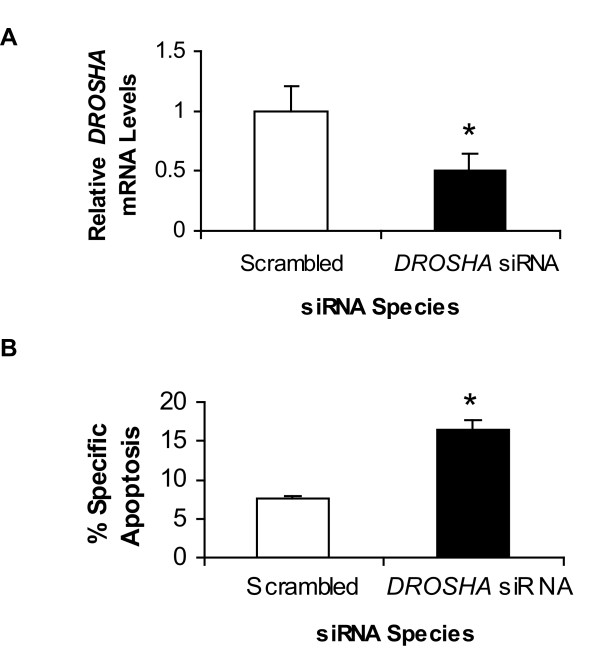
***DROSHA *down-regulation sensitizes cells to apoptosis induced by 5-FU**. Empty vector-expressing HT29 cells were transfected with scrambled or *DROSHA *siRNA for 48 h. **(A) **Quantitative RT-PCR results confirm that *DROSHA *expression was decreased (*P *< 0.05). Results are the mean ± S.D. of 4-5 independent experiments. **(B) **Knockdown of *DROSHA *increased apoptosis in response to 5-FU, as assayed with 7-AAD and Annexin V (*P *< 0.02). Results are the mean ± S.D. of 4-5 independent experiments.

The *DROSHA *gene was shown to be down-regulated by microarray analysis (see Additional file 2, Figure 2A) and NFκB occupancy on the promoter of *DROSHA *was significantly decreased upon Par-4 overexpression in HT29 cells (Figure 3C and 3D). We explored the possibility that these two connected events may contribute to the pro-apoptotic activity of Par-4. Empty vector-expressing HT29 cells were transfected with an siRNA targeting *DROSHA *and assayed for apoptosis. qRT-PCR analysis confirmed *DROSHA *down-regulation by nearly 2-fold (Figure 7A). Correspondingly, knockdown of *DROSHA *promoted the susceptibility of cells to apoptosis by 5-FU treatment (Figure 7B). It should be noted that these same empty-vector-expressing cells were demonstrated earlier to be resistant to 5-FU-mediated apoptosis in the absence of *DROSHA *knockdown (Figure 1B).

**Figure 8:**
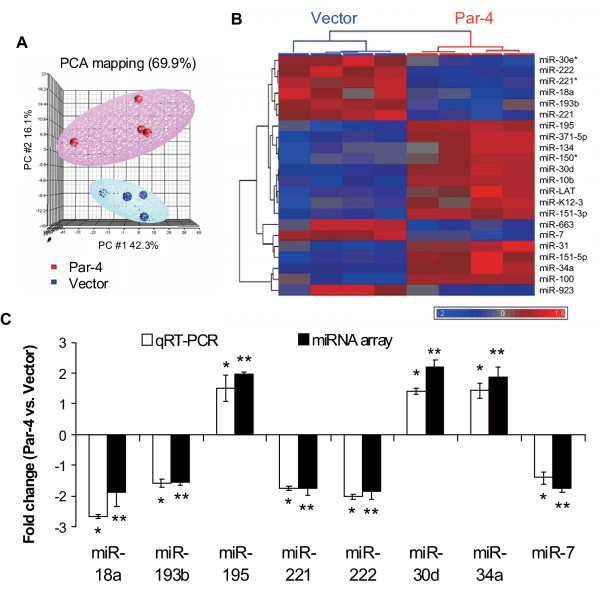
**Alterations of microRNA expression following Par-4 overexpression in HT29 cells**. **(A) **Principal component analysis (PCA) of colon cancer cells based on global microRNA expression. Three-dimensional PCA demonstrated that global microRNA expression patterns segregated (with 69.9% of the variance) in accordance with Par-4- (indicated in red) or empty vector-transfected (Vector) HT29 cells (indicated in blue). **(B) **Unsupervised hierarchical clustering of significantly up- and down-regulated microRNAs (as determined by *t*-test with 10% FDR) in Par-4- versus empty vector-transfected cells. **(C) **Validation of microRNA profiles in response to Par-4 overexpression. MicroRNAs identified as altered following Par-4 overexpression were validated by qRT-PCR. Fold changes for miR-18a, miR-193b, mi-195, miR-221, miR-222, miR-30d, miR-30d, miR-34a and miR-7 are indicated. Results are the mean ± S.D. of 4-5 independent experiments. *,** Significantly different between Par-4- and empty vector-transfected cells (*P *< 0.05).

The down-regulation of *DROSHA *suggested potential dysregulation of microRNAs. Consequently, we investigated the effects of Par-4 overexpression on global microRNA expression profiles. MicroRNA expression data from four independent Par-4-transfected cell lines and four independent empty vector-transfected cell lines were subjected to unsupervised principal components analysis (PCA). Principal components 1, 2 and 3, accounting for 69.9% of the variation in 180 expressed microRNAs, showed clear separation between cell lines with and without Par-4 overexpression (Figure 8A). A student's *t*-test (double-sided) with multiple test correction of 10% FDR revealed 22 microRNAs were significantly differentially expressed between Par-4 and empty vector-transfected cells (Additional file 5). Among the 22 differentially expressed microRNAs, 13 microRNAs were up-regulated (e.g. miR-34a, miR-100) and 9 microRNAs were down-regulated (e.g. miR-221, miR-222) by Par-4. Unsupervised hierarchical clustering based on the 22 microRNAs confirmed the segregation between empty vector- and Par-4-transfected cells (Figure 8B). Lastly, siRNA-mediated knockdown of *DROSHA *in HT29 cells (thus bypassing Par-4 overexpression) was found to up-regulate miR-34a levels by more than 2-fold compared to scrambled control as defined by qRT-PCR (*P *< 0.05, unpaired t-test; n = 4 independent determinations). This finding confirms a direct link between changes in *DROSHA *expression and microRNA profiles.

### Genes predicted to be targeted by deregulated microRNAs are associated with cell death pathways

TargetScan 4.2 was integrated into the Agilent GeneSpring GX program and used to predict the mRNAs targeted by the 22 deregulated microRNAs in Par-4-overexpressing cells. By applying strict criteria on the TargetScan algorithm, 1187 mRNAs were identified as predicted targets (Additional file 6). Subsequently, we applied Ingenuity Pathway Analysis (IPA) to ascertain potential gene networks, diseases, molecular functions, and canonical pathways associated with the predicted target mRNAs. Cancer, cell death, cell morphology, and gene expression were identified as the top gene networks. Among the set of 1187 target mRNAs, 283, 273, 150 and 244 target mRNAs were functionally associated with cell death, cell growth/proliferation, cell cycle and gene expression, respectively. IPA results also revealed target mRNAs involved in the WNT/β-catenin (*P *= 2.37 × 10^-6^), ERK/MAPK (*P *= 6.96 × 10^-6^) and PI3K/AKT canonical signaling pathways (*P *= 8.31 × 10^-6^) (Additional file 7).

Eleven (miR-30d, miR-10b, miR-34a, miR-195, miR-222, miR-221, miR-31, miR-7, miR-663, miR-193b and miR-18a) out of 22 deregulated microRNAs accounted for the 283 predicted target mRNAs linked to cell death (e.g. pro- or anti-apoptotic genes) (see Additional file 8). Among these eleven apoptosis-associated microRNAs, eight were selected for validation by qRT-PCR assays. The expression levels of individual microRNAs were normalized with miR-103 expression, one of the most stable "house-keeping" microRNA species in most human tissues [39]. Our qRT-PCR results showed that miR-18a, miR-193, miR-221, miR-222 and miR-7 were down-regulated, whereas miR-195, miR-30d and miR-34a were up-regulated in Par-4-transfected cells when compared with empty vector-transfected cells (Figure 8C). The fold change values from qRT-PCR results were in agreement with the results derived from Agilent microRNA microarrays (Figure 8C).

### Functional validation of predicted microRNA targets: miR-34 inhibits BCL2 protein expression and induces apoptosis in Par-4-overexpressing cells

**Figure 9:**
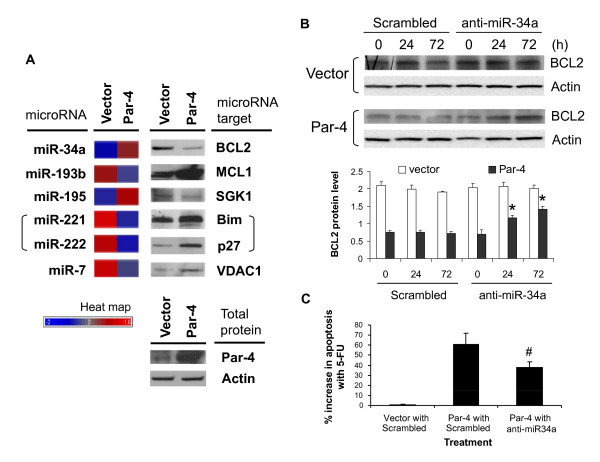
**Par-4 overexpression modulates translation of apoptosis-associated genes and sensitizes HT29 cells to 5-FU-mediated apoptosis via miR-34a regulation of *BCL2 *expression**. (**A**) Post-translational regulation of apoptosis and anti-apoptosis gene expression by microRNAs upon Par-4 overexpression. Western analysis was performed to assess levels of anti-apoptotic proteins (BCL2, MCL1 and SGK1) and apoptotic proteins (Bim, p27 and VDAC1) targeted by the corresponding differentially expressed microRNAs in empty vector- and Par-4-transfected cells. Note that both miR-221 and miR-222 are predicted to target *BCL2L11 *(encoding Bim) and *CDKN1B *(p27). Heat map represents the relative microRNA expression levels between empty vector- and Par-4-transfected cells. **(B) **Western of BCL2 in empty vector- and Par-4-transfected cells treated with or without miR-34a antagomir. Par-4-overexpressing and empty vector-expressing cells were transfected with scrambled or miR-34a antagomir. Transfected cells were harvested at 0, 24, and 72 h to assess the effect of inhibiting miR-34a on BCL2 protein levels. Quantification of relative BCL2 protein levels were calculated from BCL2-to-actin ratios. Results are the mean ± S.D. of 3-4 independent experiments. *Significantly different from 0 h following miR-34a antagomir treatment (*P *< 0.05). **(C) **Inhibition of miR-34a in Par-4-overexpressing HT29 cells reverses apoptotic sensitization to 5-FU treatment. Empty vector-expressing cells transfected with scrambled antagomir (Vector with Scrambled), Par-4-overexpressing cells transfected with scrambled antagomir (Par-4 with Scrambled) and Par-4-overexpressing cells transfected with miR34a antagomir (Par-4 with anti-miR-34a) for 24 h were treated with 100 μM 5-FU for an additional 24 h and assayed for apoptosis. Results are the mean ± S.D. of 4-5 independent experiments. ^#^Significantly different from Par-4 with Scrambled (*P *< 0.05).

We sought to validate 6 TargetScan predictions from above. MiR-34a and miR-195, both up-regulated by Par-4 overexpression, were predicted to target the pro-survival genes *BCL2 *and *SGK1*, respectively (Figure 8B and 8C; see Additional file 8). The Par-4-down-regulated microRNAs miR-221, miR-222 and miR-7 were predicted to target the pro-apoptotic genes *BCL2L11 *(*Bim*), *CDN1B *(*p27*) and *VDAC1*, respectively (Figure 8B and 8C; see Additional file 8). Finally, the Par-4-down-regulated microRNA miR-193b was predicted to target the pro-survival gene *MCL1*. None of the predicted targets were shown by mRNA expression profiling to be differentially regulated by Par-4 overexpression (Additional file 2). To the best of our knowledge, the predicted target mRNAs of miR-193b, miR-195 and miR-7 have yet to be functionally validated; whereas *BCL2*, *BCL2L11 *(*Bim*) and *CDN1B *(*p 27*) have been functionally validated (by Western blot or qRT-PCR analysis) as target mRNAs of miR-34a, miR-221 and miR-222 in human neuroblastoma, prostate cancer and rat PC12 cell lines [40-42]. Our Western blot analysis revealed a negative correlation between microRNA and target gene expression (Figure 9A). For example, the up-regulation of miR-34a and miR-195 was associated with a down-regulation of BCL2 and SGK1 protein levels, respectively; whereas the down-regulation of miR-193b, miR-221, miR-222 and miR-7 was associated with an up-regulation of MCL1, BCL2L11 (Bim), CDN1B (p 27) and VDAC1 protein levels, respectively (Figure 9A). These results demonstrate perfect concordance between microRNA expression changes and protein level changes corresponding to the target predictions.

Next, the functional relevance of one validated microRNA/target mRNA pair in the sensitization of HT29 cells to apoptosis by 5-FU was explored in greater detail. Evidence has implicated miR-34a as a crucial component of the p53 tumor suppressor network with potent anti-proliferative and pro-apoptotic activity [43-45]. In addition, *BCL2 *mRNA was recently identified as a true miR-34a target in neuroblastoma cell lines [40]. Here, we hypothesized that up-regulation of miR-34a via *DROSHA *deregulation causes repression of BCL2 protein expression, thus contributing to the initiation of apoptotic sensitivity in Par-4-overexpressing cells. To more directly assess the role of miR-34a in the regulation of BCL2 expression and induction of apoptosis, Par-4-overexpressing HT29 cells were transfected with a chemically modified single-strand RNA antagomir, complementary to miR-34a, to block miR-34a function. As shown in Figure 9B, BCL2 protein levels of Par-4-overexpressing cells were substantially lower than empty vector-transfected cells at time 0 h. However, subsequent transfection of Par-4-overexpressing cells with the miR-34a antagomir gradually increased BCL2 protein levels at 24 h and 72 h, when compared with negative control scrambled oligonucleotide transfected cells (Figure 9B, bottom panel). In contrast, no significant changes in BCL2 protein levels were detected at 0, 24, or 72 h in the empty vector-transfected cells with or without miR-34a inhibition (Figure 9B, bottom panel). Finally, apoptotic sensitivity to 5-FU decreased in Par-4-overexpressing cells transfected with anti-miR-34a (Figure 9C). These results suggest that up-regulated miR-34a is involved in both the repression of BCL2 protein expression and increased 5-FU sensitivity in Par-4-overexpressing cells.

## Discussion

Par-4 plays an important role in activating intrinsic pro-apoptotic signaling pathways [11]. Consequently, Par-4 has gained interest as a potential modality for molecular therapy since it has been reputed to induce apoptosis exclusively in cancer cells but not normal cells [10]. We have demonstrated that *PAR-4 *mRNA levels are significantly decreased nearly 3-fold in colon cancer patient samples relative to their paired normal colon. While in some cells the increase in Par-4 alone is sufficient to cause cell death [19,24,25], the ectopic introduction of Par-4 into HT29 colon cancer cells did not induce apoptosis but rather heightened cell sensitivity to the apoptotic stimulus of 5-FU. Likewise, Par-4 has been shown to sensitize neoplastic lymphocytes to apoptotic stimuli such as TRAIL and CD95 [21,26]. Our study also has uncovered a novel binding partnership between Par-4 and NFκB. In HT29 and SW480 cells, pharmacologic (ISC-4) or genetic (pshAkt1) suppression of AKT1 activity resulted in increased Par-4/NFκB and decreased Par-4/14-3-3 interactions. The latter finding is in line with previous results demonstrating that binding of Par-4 to 14-3-3 is dependent on AKT1 activity [19]. Additional partner proteins of Par-4 include ζPKC [30], TOP1 [46], WT1 [47] and ZIP kinase [48]. Par-4/ζPKC interactions in the cytoplasm of NIH3T3 fibroblasts [27,30], and Par-4/TOP1 interactions in the nucleus of immortalized epithelial cells impede NFκB transcriptional activity [46]. Our findings in colon cancer cells are consistent with an alternative and possibly complimentary pathway for the modulation of NFκB transcriptional activity via direct Par-4/NFκB interactions in the cytoplasm. Support for this alternative mechanism is based on the observations that Par-4 overexpression increased Par-4/NFκB partnerships almost exclusively in the cytoplasm (confocal colocalization microscopy, subcellular fractionation and co-immunoprecipitation), repressed NFκB gene transcription (luciferase reporter assay), inhibited nuclear translocation (subcellular fractionation and Western, confocal colocalization microscopy), repressed NFκB binding to *cis*-binding sites in a number of pro-survival and anti-apoptotic genes (ChIP-qPCR), and affected the expression of genes primarily with NFκB binding sites in their promoters (DNA microarrays, position weight matrix similarity analysis). Hence, it appears that inhibition of NFκB translocation by Par-4 can occur through partnerships with ζPKC [27] and/or the NFκB/IκB complex (present study).

One of the more notable NFκB target genes down-regulated by Par-4 was *DROSHA*, which encodes a nuclear RNase III enzyme responsible for the processing of microRNAs [49]. Knockdown of *DROSHA *by siRNA resulted in increased apoptotic responsiveness of colon cancer cells to 5-FU, portending a potential role of the microRNA pathway in Par-4-mediated apoptotic sensitivity. MicroRNAs are important in the regulation of crucial biological processes and alterations in microRNA expression are proposed to play a role in the pathophysiology of many, perhaps, all human cancers [50,51]. Our findings suggest that *DROSHA *down-regulation, in response to Par-4 overexpression, disturbs global microRNA biogenesis, and that deregulation of microRNA expression will have consequential widespread effects on the post-transcriptional regulation of genes (mRNAs targeted by microRNAs). It is unclear at this time why Par-4-mediated down-regulation of *DROSHA *in HT29 cells would be associated with both a down- and up-regulation of microRNAs. Notwithstanding, overexpression or recruitment of DROSHA has been shown both to down- and up-regulate microRNAs in carcinoma samples [52,53]. These results suggest that a complex regulatory circuit exists between NFκB activity and *DROSHA *regulation of microRNA biogenesis in colon cancer cells.

The functional consequences of *DROSHA *down-regulation and associated microRNA deregulation in Par-4-overexpressing HT29 cells was assessed computationally. Sixty percent of the predicted target mRNAs of the deregulated microRNAs appear to be associated with apoptosis, cell proliferation and cell cycle regulation. We have successfully validated a subset of these predictions by Western blot analysis. Moreover, one particular deregulated microRNA miR-34a, which was up-regulated in response to overexpressed Par-4, was functionally characterized in greater detail. Inhibition of miR-34a in Par-4-overexpressing cells resulted in an up-regulation of BCL2 protein with a corresponding decrease in apoptotic sensitivity to 5-FU. It should be noted that a primary transcript containing miR-34a can be directly transactivated by p53 [43,45]. Our findings support an alternate indirect pathway, involving Par-4/NFκB/*DROSHA*, that promotes apoptosis. Interestingly, Cheema et al. reported on a pathway that directly regulates BCL expression via Par-4 interactions with transcription factor WT1 at the *BCL2 *gene promoter in prostate cancer cell lines [35]. We did not observe changes in *BCL2 *mRNA levels in Par-4-overexpressing HT29 cells, suggesting that the primary mechanism of down-regulating BCL2 protein was post-transcriptional.

A number of Par-4/NFκB/*DROSHA*-regulated microRNAs identified in this study have been reported to be associated with tumorigenesis in patients. For example, miR-221, and miR-222 are up-regulated, while miR-34a, miR-18a, miR-30d and miR-34b are down-regulated in colon cancer [54-56]. Moreover, miR-221, miR-222 and miR-134 are up-regulated in lymphocytic leukemia, pancreatic, liver, esophagus, or thyroid cancers [57-59], whereas miR-34a and miR-100 are down-regulated in neuroblastoma [60], esophagus and ovary cancers [8,58]. Of interest, the direction of regulation for many microRNAs, including miR-34a, in patient tumors is consistent with our *in vitro *cell line model.

## Conclusions

In conclusion, Par-4 may play a significant role in the treatment of colon cancer by increasing the sensitivity of colon cancer cells to undergo apoptosis through the binding of Par-4 to NFκB in the cytoplasm, thus inhibiting translocation of NFκB to the nucleus and altering both *DROSHA *and microRNA expression. Accordingly, enhancing Par-4 activity and/or modifying the expression of microRNA processing genes or microRNAs themselves may provide an effective strategy for the treatment of colon cancer.

## Methods

### Cell culture

Human colon cancer cells, SW480 and HT29 (American Type Culture Collection, Manassas, VA), were cultured in RPMI (Cellgro, Mediatech, Inc, Manassas, VA) containing 10% FBS and Pen/Strep at 37°C and 5% CO_2_. Both SW480 and HT29 cells are part of the NCI-60 panel of cancer cell lines and represent two colon cancer lines with a wealth of biochemical, molecular, proteomic and genomics data, providing an opportunity for meta-analysis [61-63]. Cells were transfected with either rat *par-4 *cDNA in pCB6+ or with empty vector using Fugene 6 reagent (Roche Diagnostics, Indianapolis, IN, USA). Transfectants were selected with G418 (Gibco, Carlsbad, CA) and colonies expanded and assayed for Par-4 expression. HT29 cells were transfected with 1 μg/ml *DROSHA *siRNA or the corresponding scrambled siRNA (Thermo Scientific Dharmacon, Lafayette, CO) using Lipofectamine 2000 (Invitrogen, Carlsbad, CA).

### Subcellular fractionation, immunoprecipitation and Western blotting

Antibodies used were Par-4, NFκB p 50, p 65, BCL2, MCL1, BCL2L11 rabbit polyclonal, SGK1, VDAC1 goat polyclonal, CDN1B (p27) mouse monoclonal (Santa Cruz, Santa Cruz, CA, USA), AKT1 mouse monoclonal (Cell Signaling, Danvers, MA, USA), PKA goat polyclonal, PARP rabbit polyclonal (Upstate Cell Signaling Solutions, Charlottesville, VA, USA), and β-actin mouse monoclonal (Sigma, Saint Louis, MO, USA). Subcellular fractionations were performed using the NE-PER Nuclear and Cytoplasmic extraction reagent kit (Pierce Biotechnology, Rockford, IL, USA) according to the manufacturer's instructions. Western blotting and immunoprecipitation assays were performed as previously documented [64]

### Synthesis of ISC-4

ISC-4 was synthesized following a method recently developed by Sharma *et al. *[65] Briefly, a solution of triphosgene (1.48 g, 5.0 mmol) in CH_2_Cl_2 _(15 mL) was added dropwise, for a period of 1 h, to a refluxing mixture of phenylbutyl formamide (1.77 g, 10.0 mmol), triethylamine (4.35 g, 6.0 mL, 43.0 mmol) and 4Å molecular sieves in CH_2_Cl_2 _(50 mL). The mixture was refluxed for an additional 2.5 h. Selenium powder (1.58 g, 20 mmol) was added and the resulting mixture was refluxed for additional 7 h. The mixture was purified by silica gel column chromatography (EtOAc/hexanes 5:95) to yield 1.7 g (71%) of ISC-4 as viscous oil.

### Assays of apoptosis

For caspase-3 activity assay, cells were cultured in 6-well plates with half of the wells treated with 100 μM 5-FU. After 24 hrs cells were harvested and assayed for apoptosis using the Caspase-3 Assay kit from BD Biosciences Pharmingen (San Diego, CA). For annexinV/7AAD assay, cells were cultured in a 12 well plate and treated as above. Cells were trypsinized, washed, and stained with Annexin V, conjugated with PE, and 7AAD (BD Biosciences, San Jose, CA). Staining was detected by flow cytometry. MTS assay was performed in 96 well plates according to manufacturer's protocol (Promega Corp, Madison, WI). After addition of MTS solution, plates were incubated at 37° for 4 h and read at 490 nm using a plate reader.

### Treatment with AKT inhibitors

HT29 cells were treated with 3 to 50 μM ISC-4 for 48 hours. *In vitro *cytotoxic efficacy was measured using 3-(4,5-dimethylthiazol-2-yl)-5-(3-carboxymethoxyphenyl)-2-(4-sulfophenyl)-2H-tetrazolium (MTS) viability assay (Promega, Madison, WI, USA).

### NFκB reporter activity assay

NFκB activity was assessed using the Cignal™ NFκB Reporter Assay Kit (SuperArray, Bioscience Corporation, Frederick, MD, USA). Cells were transfected with 6.6 μg/ml of the reporter plasmids. Cells were incubated 48 hours, and assayed using the Dual-Luciferase Reporter Assay System (Promega Corporation, Madison, WI, USA). Cells were lysed, proteins quantitated, and 26 μg protein added to each well of a 96-well opaque white plate. The plate was read in a Synergy plate reader, with KC4 software (Bio-Tek Instruments, Winooski, VT, USA). Firefly luciferase substrate was injected to assess luciferase activity under the control of the NFκB promoter. Renilla luciferase substrate was added to assess transfection efficiency. Each well was read 11 times after a 2 second delay over a period of 10 seconds.

### Gene expression profiling analysis

Gene (mRNA) expression profiling experiments were performed on a 39,936 human cDNA microarray employing a common reference design as previously described [66,67]. LOWESS data normalization, experimental noise determination, and statistical analysis with false discovery rate (FDR) to correct for multiple testing were performed as described previously [66-68]. Biological themes associated with the differentially expressed genes were identified using gene ontology (GO) categories in the Expression Analysis Systematic Explorer (EASE) application [69], which is executable using TIGR Multi Experiment Viewer (TMEV; available at http://www.tigr.org/softlab). A Fisher's Exact score p < 0.05 was considered significant.

### Real-time RT-PCR validation of mRNA expression

Quantitative RT-PCR assay was performed on the ABI 7900 HT Sequence Detection System using Assay on Demand primers and probes and TaqMan Universal PCR Master Mix (Applied Biosystems, Foster City, CA). PCR conditions were 2 minutes at 50°C, 10 minutes at 95°C and 40 cycles of 15 seconds at 95°C and 1 minute at 60°C. ABI SDS 2.2.2 software and the 2^-ΔΔCt ^analysis method [70] were used to quantitate relative amounts of product using beta-actin as an endogenous control.

### Promoter analysis for NFκB binding sites

For each differentially regulated gene identified by microarray analysis, 5,000 bases of the proximal promoter region (-5,000 to -1) were extracted from ENSEMBL database http://www.ensembl.org. Position weight matrix (PWM) models representing the binding sites for NF-κB family members p50 and p65 were taken from version 7.0 of the TRANSFAC database [71], using matrix IDs 'V$NFKAPPAB50_01' and 'V$NFKAPPAB65_01', respectively. Matches to each PWM were identified in promoter regions using a slightly modified version of *tffind *[72], with default matrix similarity thresholds.

### Chromatin immunoprecipitation analysis

Chromatin immunoprecipitation (ChIP) reagents were purchased from Upstate Cell Signaling (Billerica, MA, USA). HT29 colon cancer cells (1 × 10^6^) were transfected with empty vector or Par-4 expressing vector and treated with 1% formaldehyde for 15 min at 37°C to crosslink protein-chromatin complexes. The fixed cells were washed twice with cold PBS, and chromatin DNA was harvested and sonicated for ChIP assays. The ChIP assays were performed according to the manufacturer's protocol. Anti-NFκB p50 and p65 antibodies used for ChIP assays were from Abcam (Cambridge, MA, USA) and Santa Cruz (Santa Cruz, CA, USA), respectively. In ChIP experiments, quantitative real-time PCR (qPCR) analysis with SYBR Green PCR Master Mix (Applied Biosystems, Foster City, CA, USA) were calculated by measuring the ratios of ChIP-to-Input, and the non-antibody-treated chromatin immunoprecipitated samples were used as a negative control. NFκB occupancy at *IL8 *and *ACTB *promoters served as additional positive and negative controls, respectively, for the ChIP-qPCR experiments. All primers used for quantitative PCR are listed in Supplemental data (Additional file 9).

### Immunocytochemistry with confocal fluorescence detection

Empty vector-transfected HT29 cells, Par-4-overexpressing HT29 cells, and native HT29 cells transfected with scrambled or pshAkt1 were grown in 6-well plates on glass coverslips. After culturing for 24 h (empty vector- and Par-4-transfected cells) or 48 h (scrambled- and pshAkt1-transfected cells), cells were fixed with 3.7% formaldehyde in phosphate-buffered saline (1×PBS) for 15 min at room temperature and washed once with 1×PBST (1×PBS with 0.1% Tween-20). Cells were permeabilized with 0.1% Triton X-100 in 1×PBS for 5 min followed by two washes with 1×PBST. The cells were then treated with blocking buffer (5% FBS in 1×PBS) for 1 h, and incubated with the rabbit polyclonal anti-NF-κB p65, Par-4 (Santa Cruz Biotechnology, Santa Cruz, CA, USA) or p105/p50 (Abcam, Cambridge, MA, USA) antibody, or the mouse monoclonal anti-Par-4 or IκB-antibody (Santa Cruz Biotechnology, Santa Cruz, CA, USA) at 1:400 dilution for overnight at 4°C, followed by two washes with 1×PBST. For the secondary antibodies, Alexa Fluor 488 donkey anti-rabbit IgG and Alexa Fluor 594 goat anti-mouse IgG (Molecular Probe, Carlsbad, CA, USA) were used at 1:1000 dilutions and incubated for 1 h at room temperature in the dark, and then washed three times with 1×PBST. Coverslips were mounted onto glass slides with Prolong Gold antifade reagent with DAPI (Molecular Probe, Carlsbad, CA, USA) to detect the cell nuclei. Confocal microscopy was performed with a Zeiss Axioplan fluorescence microscope coupled with a Zeiss LSM710 Laser Scanning System (Zeiss, Berlin, Germany). Colocalization analysis was performed using Volocity 5.0 software (PerkinElmer, Waltham, MA, USA). Positive PDM (product of the difference from the mean) channels were generated to visualize the highly-correlated colocalization of two fluorescence-labeled proteins (Figure 5A-v, B-v and 5B-x). Images were processed with Volocity software (PerkinElmer, Waltham, MA, USA).

### Recurrent co-regulated gene network analysis

Recurrent co-regulated (ReCo) gene networks were constructed from 334 colon cancer gene expression microarray studies downloaded from the Expression Project for Oncology repository https://expo.intgen.org/geo/listPublicGeoTransactions.do and Gene Expression Omnibus (GEO) [73]. In a separate analysis, we also inspected the ReCo links in prostate-disease networks given that Par-4 is known to have an important role in prostate cancer. Using only data corresponding to a specific tissue and disease state (i.e. colon cancer or prostate cancer), a co-regulated network was constructed by linking gene pairs that were significantly coexpressed across the corresponding samples. Network analysis was restricted to the set of genes initially identified as Par-4-targets containing NFκB binding sites in colon cancer cell lines. A Pearson correlation *r *was calculated for all pairs of genes (i, j):

where *N *is the number of samples, *E*_*is *_is the expression level of gene *i *in sample *s*, *μ*_*i *_is the mean expression level of gene *i *across all samples, and *σ*_*i *_is the standard deviation of the expression level of gene *i*.

Pearson correlations were then transformed to *Z*-scores using the Fisher *Z *transformation:

Intuitively, the Fisher *Z *transformation assigns higher significance to genes with strong correlations across a greater number of samples. *Zij*'s can be interpreted as Z-scores which provides an estimate of the significance of two gene's correlation across the set of conditions. While the Z-score would be exact if the expression levels across the conditions were independent and normally distributed, which is clearly not true in our case, it still provides a good measure of relative correlation for a single experiment useful for ranking gene pairs against one another.

We calculated a tissue-disease-specific ReCo network by combining the Z scores of gene pairs across individual studies of the same tissue-disease state. We first converted the *Z*-scores for each gene pair (*i, j*) in each study *S *to *P *values using the following rank ratio transformation:

where *L *is the total number of gene pairs in the dataset. Then, the *P *values were combined across studies using the Mudhalker-George's *t*-statistic to derive a *recurrent co-regulated (RECO) *score:

where *N*_*s *_is the total number of studies. The resulting *RECO *score gives a single value quantifying the strength of coexpression of each gene pair (*i*, *j*) across all available studies of the same tissue-disease state.

### microRNA microarray hybridization and data analysis

Total RNA from HT29 cells was harvested in QIAzol Lysis reagent (Qiagen, Valencia, CA, USA), and microRNA isolated using miRNeasy Mini Kit (Qiagen, Valencia, CA, USA) according to the manufacturers' instructions. RNA quality was assessed and quantified using the RNA 6000 Nano Kit (Agilent Technologies, Inc., Santa Clara, CA). microRNeasy-isolated RNA (250 ng) was used as input in the labeling reaction, and the entire reaction was hybridized onto an Agilent human genome microRNA microarray V1 (Agilent Technologies, Inc., Santa Clara, CA) containing 20-40 probes for each of 470 microRNAs for 20 hours at 55°C. Hybridization signal intensities were extracted using the Agilent Feature Extraction software. Raw mean signal, total probe intensities and total gene intensities were uploaded into GeneSpring GX 10.0 software (Agilent Technologies, Santa Clara, CA). Poor spots, as reported in the raw data file, were flagged as A (absent). The background-subtracted signal intensities were log_2 _transformed and quantile normalized. GeneSpring GX 10.0 and Partek Genomic Suite 6.2 (St. Louis, MO) were used for principal component analysis (PCA), statistical analysis, and hierarchical clustering. Differential expression was assessed using a two sample *t*-test (double-sided). Corrected *P*-values were adjusted for multiple testing using Benjamini and Hochberg's FDR at 10% [74].

### Real-time RT-PCR validation of microRNA expression

qRT-PCR was performed using the NCode™ EXPRESS SYBR^® ^GreenER™ microRNA qRT-PCR Kit (Invitrogen, Carlsbad, CA). Poly(A) tailing and RT reactions consisted of 4 μl 5× reaction mix, 2 μl 10× SuperScript enzyme mix, 0.5 mL and 200 ng total RNA in a final volume of 20 μl. Following poly(A) tailing and RT steps, 0.17 μl of the RT product was transferred into a PCR reaction mixture consisting of 10 μl Express SYBR green qPCR SuperMix, 0.4 μl microRNA-specific forward primer (10 μM), 0.4 μl universal qPCR primer (10 μM) in a final volume of 20 μl. PCR cycling began with template denaturation and hot start Taq activation at 95°C for 2 min, then 40 cycles of 95°C for 15 sec, and 60°C for 1 min performed in a 7300 Real-Time PCR System (Applied Biosystems, Foster City, CA). MiR-103 was used as the internal standard reference in the qRT-PCR reaction [39]. Normalized expression was calculated using the comparative Ct method and fold change was derived from the equation 2^-ΔΔCt ^for each microRNA.

### Identification of microRNA target genes and pathway analyses

Target Scan database (version 4.2, http://www.targetscan.org) was integrated to GeneSpring GX and used for the identification of target genes. Target Scan allows identification of target mRNAs for any specific microRNA, based on the context score percentile [75]. Differentially expressed microRNAs with 10% FDR were imported into the TargetScan algorithm and the target mRNAs with high context scores (context percentile of 80) were retained for further analysis. Predicted target mRNAs were imported to GeneSpring GX 10.0 and Ingenuity Pathway Analysis programs to identify significant biological pathways, associated network functions, and associated molecular and cellular functions.

### Inhibition of miR-34a expression

Cells grown to 60-70% confluence in 6-well plates were transfected with 150 pmole anti-miR-34a inhibitor or negative-control oligonucleotide (Ambion, Austin, TX) using Lipofectamine 2000 according to manufacturer's protocol. The medium was replaced with fresh medium after 24 hours, and cells were allowed to grow for another 48 hours prior to functional analysis.

## List of abbreviations

5-FU: 5-fluorouracil; ISC-4: phenylbutyl isoselenocyanate; Par-4: prostate apoptosis response protein-4; NFκB: nuclear factor kappaB; PKA: protein kinase A; TRAIL: tumor necrosis factor-related apoptosis-inducing ligand; TNFα: tumor necrosis factor alpha.

## Competing interests

The authors declare that they have no competing interests.

## Authors' contributions

BDW performed experiments, analyzed results, generated figures and wrote the paper; CLBK performed experiments and wrote the paper; DMP performed immunoprecipitation experiments; TLO analyzed results; BF and TL performed microarray experiments, AKS synthesized Akt inhibitor ISC-4; GR performed experiments; MTW and JMS performed promoter and recurrent coregulated gene network analyses; SRP revised the paper; NHL and RBI designed research, analyzed results, generated figures and wrote the paper. All authors read and approved the final manuscript.

## Supplementary Material

Additional file 1 Par-4 expression in colon cancer patient samples and paired normal colon. Click here for file

Additional file 2 Microarray data. Click here for file

Additional file 3 Representative biological roles of genes identified by microarray analysis of Par-4 vs. empty vector-transfected cells. Click here for file

Additional file 4 p65 and p50 binding sequences of NFκB target genes. Click here for file

Additional file 5 Differentially expressed microRNAs in HT29 cells transfected with Par-4 or empty vector. Click here for file

Additional file 6 Target mRNAs of the deregulated microRNAs (predicted by Target Scan). Click here for file

Additional file 7 Ingenuity pathway analysis (IPA) of genes predicted to be targeted by the differentially expressed microRNAs in cells with and without Par-4 overexpression. Click here for file

Additional file 8 Predicted target genes of differentially expressed microRNAs have known associations with apoptotic or anti-apoptotic function. Click here for file

Additional file 9 Primer sequences for ChIP-qPCR experiments. Click here for file
