# Supplementary material for: Prostate apoptosis response protein 4 sensitizes human colon cancer cells to chemotherapeutic 5-FU through mediation of an NFκB and microRNA network
Source: Mol Cancer. 2010 Apr 30;9:98. doi: 10.1186/1476-4598-9-98 (PMC2883962; doi:10.1186/1476-4598-9-98)
Supplement: Additional file 1 — Par-4 expression in colon cancer patient samples and paired normal colon. [file 1476-4598-9-98-S1.PDF]

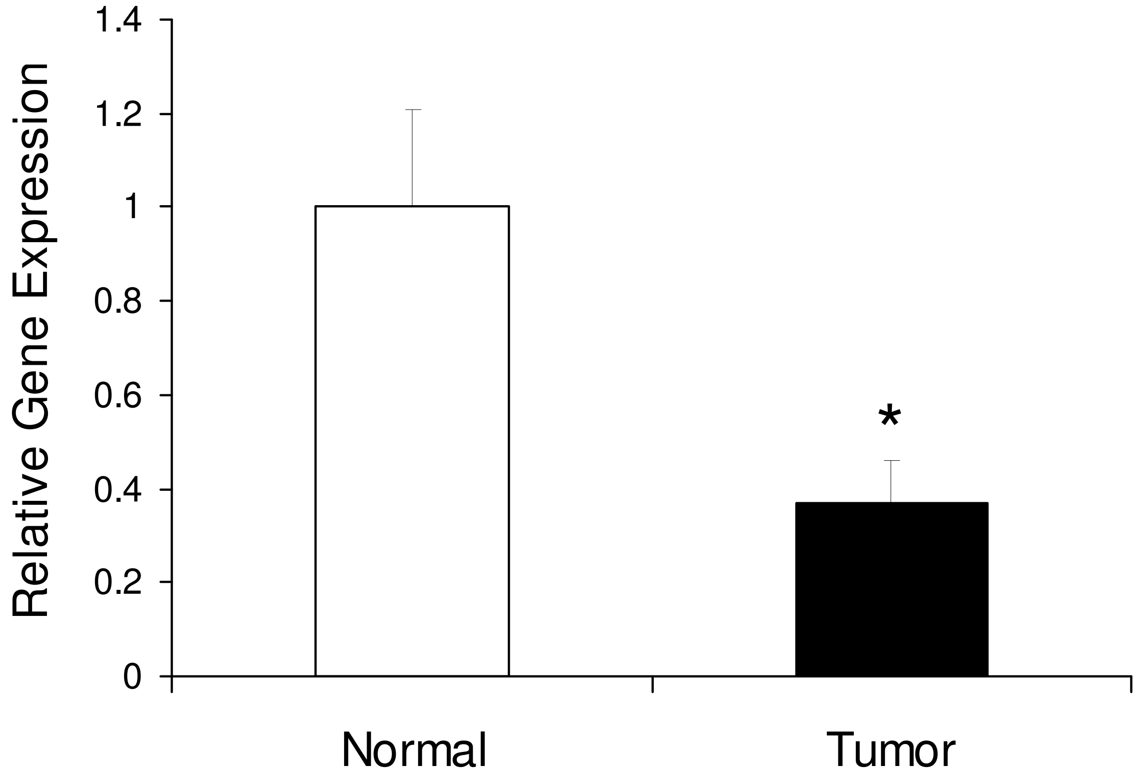

Supplemental Figure 1. *PAR-4* expression in colon cancer and paired normal colon specimens. *PAR-4* mRNA levels were examined by quantitative RT-PCR of colon cancer and paired normal sample from 11 patients. Results are the mean $\pm$ S.D. *Significantly different from paired normal colon by paired *t*-test ($P<0.05$).
